# Chest

**DOI:** 10.4103/0971-3026.38511

**Published:** 2008-02

**Authors:** Anupam Lal, Ram Prakash Galwa, Mahesha Vankalakunti, S Radhika, N Khandelwal

**Affiliations:** Department of Radiodiagnosis, Postgraduate Institute of Medical Education and Research (PGIMER), Chandigarh - 160 012, India; 1 Department of Cytology, Postgraduate Institute of Medical Education and Research (PGIMER), Chandigarh - 160 012, India

A 60-year-old woman presented with a history of pain in the back of the chest, with occasional cough, for the last 3 months. She had a history of a radical mastectomy for carcinoma of the right breast 17 years ago. The routine blood counts were within normal limits.

The patient underwent a contrast-enhanced CT scan of the chest [Figure 1A and B] and abdomen. A fine needle aspiration cytology (FNAC), using a 22-gauge spinal needle, was performed under CT guidance, which confirmed the diagnosis.

**Figure 1 (A, B):**
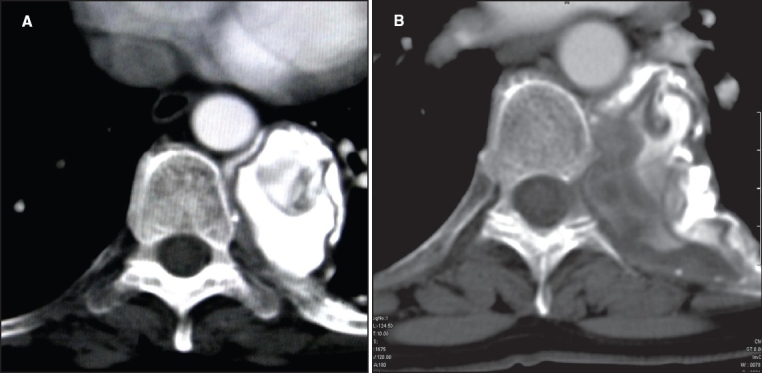
Axial contrast-enhanced CT of the chest

## What is the Diagnosis?

### Diagnosis: Calcified Left Paravertebral Hydatid Cyst

**Figure 2 (A, B):**
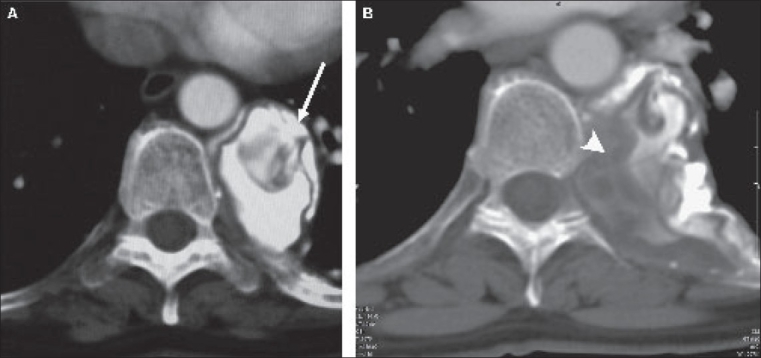
Axial contrast-enhanced CT of the chest showing a predominantly calcified mass in the left paravertebral region (arrow in A) with a noncalcified portion showing a few thin septae (arrowheads in B) representing daughter cysts

The contrast-enhanced CT of the chest [Figure 2] shows a 3.5 × 4 × 5 cm, predominantly calcified, well-defined mass in the left paravertebral region, with a noncalcified portion showing a few thin septae representing daughter cysts. Mild erosion of the neighboring 8^th^ rib is seen. The adjacent neural foramen is not widened. The lungs and abdomen were normal on the other images.

The provisional diagnosis of a calcified paravertebral hydatid cyst was offered, with alternative diagnoses of a calcified neurogenic tumour or a calcifying fibrous pseudotumor (CFPT) of the pleura. To rule out a malignant process an FNAC was performed, which confirmed the diagnosis of a hydatid cyst by demonstrating scolicial hooklets and laminated hydatid wall membranes.

Mediastinal involvement by echinococcosis is rare and only around 100 cases have been described.[1] Mediastinal hydatids are cystic lesions and are usually only incidental findings. These cysts may be oval, spherical, tubular, or dumbbell-shaped and may show intraspinal extension. Calcification in pulmonary hydatid cysts is very rare, but mediastinal hydatids tend to calcify more often than intrapulmonary lesions.[2] Benzarti *et al*. has reported a similar case of a calcified paravertebral hydatid cyst.[2]

Thameur *et al*. found mediastinal hydatid cysts in 8 of 1619 cases of intrathoracic hydatid cysts (0.5%).[3] The most common location of a mediastinal hydatid is in the thymus.[3,4]

Rakower and Milwidsky[5] recorded more than 23,000 patients with hydatid disease in various large series; only 25 cases (0.1%) were reported in the mediastinum and the paravertebral sulcus. Mediastinal hydatids are usually symptomatic; the symptoms being due to compression of adjacent organs such as the esophagus, heart, trachea, and the great vessels. Intraspinal extension may cause neurologic symptoms due to cord compression.[6] This patient's symptoms were probably due to compression of the adjacent intercostal nerves.

When calcification is present in a hydatid cyst, it can be curvilinear, lamellated, or diffuse, the curvilinear form being more common.[2] In the presence of diffuse calcifications, other calcified masses such as osteochondroma, neurogenic tumors, fibrous dysplasia, calcified hematoma or abscess, CFPT of pleura, and Castleman's disease are important differential diagnoses.[7]

Hydatids are diagnosed on the basis of the clinical, radiological, and laboratory findings, though FNAC or surgical pathology is often required for confirmation. FNAC has generally not been recommended as a diagnostic procedure since it has been believed that it could cause an anaphylactic reaction and possible dissemination of infection.[8] However studies have now shown that FNAC is a safe diagnostic procedure in the evaluation of suspected hydatid disease.[9] In our case, FNAC clinched the diagnosis by showing hydatid hooklets, with no untoward complications.

Primary treatment of mediastinal hydatids is complete surgical resection.[5] Preoperative medical treatment should be considered in order to sterilize the cyst and to decrease the tension in the cyst, thus reducing the chances of spillage and resultant anaphylaxis.[10] Postoperative medical treatment reduces the chances of recurrence.
